# Low-Coverage Whole Genome Sequencing of Cell-Free DNA From Immunosuppressed Cancer Patients Enables Tumor Fraction Determination and Reveals Relevant Copy Number Alterations

**DOI:** 10.3389/fcell.2021.661272

**Published:** 2021-08-03

**Authors:** Amira Bouzidi, Karim Labreche, Marine Baron, Marianne Veyri, Jérôme Alexandre Denis, Mehdi Touat, Marc Sanson, Frédéric Davi, Erell Guillerm, Stéphanie Jouannet, Frédéric Charlotte, Franck Bielle, Sylvain Choquet, Pierre-Yves Boëlle, Jacques Cadranel, Véronique Leblond, Brigitte Autran, Jean-Marc Lacorte, Jean-Philippe Spano, Florence Coulet, the IDEATION study group, Ahmed Idbaih

**Affiliations:** ^1^Sorbonne University, INSERM, Research Unit on Cardiovascular and Metabolic Disease UMR ICAN, Department of Endocrine Biochemistry and Oncology, AP-HP, Hôpital Pitié Salpêtrière, Paris, France; ^2^Sorbonne University, INSERM, Pierre Louis Institute of Epidemiology and Public Health, Paris, France; ^3^Sorbonne University, Center for Immunology and Infectious Diseases (CIMI-Paris), Department of Hematology, APHP, Hôpital Pitié Salpêtrière, Paris, France; ^4^Sorbonne University, INSERM, Pierre Louis Institute of Epidemiology and Public Health, Theravir Team, Medical Oncology, AP-HP, Hôpital Pitié Salpêtrière, Paris, France; ^5^Sorbonne University, INSERM, Saint-Antoine Research Center, Cancer Biology and Therapeutics, CRSA, Department of Endocrine Biochemistry and Oncology, AP-HP, Hôpital Pitié Salpêtrière, Paris, France; ^6^Sorbonne University, INSERM, CNRS, Brain and Spine Institute, ICM, Department of Neurology 2-Mazarin, AP-HP, Hôpital Pitié Salpêtrière, Paris, France; ^7^Sorbonne University, INSERM, Centre de Recherche des Cordeliers, Department of Biological Hematology, AP-HP, Hôpital Pitié Salpêtrière, Paris, France; ^8^Sorbonne University, INSERM, Saint-Antoine Research Center, Microsatellites Instability and Cancer, CRSA, Genetics Department, AP-HP, Hôpital Pitié Salpêtrière, Paris, France; ^9^Sorbonne University, Neurosurgery Department, AP-HP, Hôpital Pitié Salpêtrière, Paris, France; ^10^Sorbonne University, Anatomy and Pathologic Cytology, AP-HP, Hôpital Pitié Salpêtrière, Paris, France; ^11^Sorbonne University, Neuropathology Department, AP-HP, Hôpital Pitié Salpêtrière, Paris, France; ^12^Sorbonne University, Chest Department and Thoracic Oncology, GRC 04, Theranoscan, AP-HP, Hôpital Tenon, Paris, France; ^13^Sorbonne University, INSERM, CNRS, Center for Immunology and Infectious Diseases (CIMI-Paris), AP-HP, Pitié-Salpêtrière Hospital, Paris, France

**Keywords:** liquid biopsy, cfDNA, CNA profile, cancer, immunodeficiency

## Abstract

Cell-free DNA (cfDNA) analysis is a minimally invasive method that can be used to detect genomic abnormalities by directly testing a blood sample. This method is particularly useful for immunosuppressed patients, who are at high risk of complications from tissue biopsy. The cfDNA tumor fraction (TF) varies greatly across cancer type and between patients. Thus, the detection of molecular alterations is highly dependent on the circulating TF. In our study, we aimed to calculate the TF and characterize the copy number aberration (CNA) profile of cfDNA from patients with rare malignancies occurring in immunosuppressed environments or immune-privileged sites. To accomplish this, we recruited 36 patients: 19 patients with non-Hodgkin lymphoma (NHL) who were either human immunodeficiency virus (HIV)-positive or organ transplant recipients, 5 HIV-positive lung cancer patients, and 12 patients with glioma. cfDNA was extracted from the patients’ plasma and sequenced using low-coverage whole genome sequencing (LC-WGS). The cfDNA TF was then calculated using the ichorCNA bioinformatic algorithm, based on the CNA profile. In parallel, we performed whole exome sequencing of patient tumor tissue and cfDNA samples with detectable TFs. We detected a cfDNA TF in 29% of immune-suppressed patients (one patient with lung cancer and six with systemic NHL), with a TF range from 8 to 70%. In these patients, the events detected in the CNA profile of cfDNA are well-known events associated with NHL and lung cancer. Moreover, cfDNA CNA profile correlated with the CNA profile of matched tumor tissue. No tumor-derived cfDNA was detected in the glioma patients. Our study shows that tumor genetic content is detectable in cfDNA from immunosuppressed patients with advanced NHL or lung cancer. LC-WGS is a time- and cost-effective method that can help select an appropriate strategy for performing extensive molecular analysis of cfDNA. This technique also enables characterization of CNAs in cfDNA when sufficient tumor content is available. Hence, this approach can be used to collect useful molecular information that is relevant to patient care.

## Introduction

Patients infected with human immunodeficiency virus (HIV) or who are organ transplant recipients are at increased risk of developing several cancers compared to the general population (Hortlund et al., 2017; Yarchoan and Uldrick, 2018). Moreover, the risk of cancer-related mortality is increased in these patients (Sigel et al., 2011; Au et al., 2019; Horner et al., 2020). Tissue biopsies performed on immunosuppressed patients entails an elevated risk of complications and demonstrates lower diagnostic yields and increased mortality (Plattner et al., 2018; Cleveland et al., 2020). Compared with tissue biopsy, liquid biopsy such as cell-free DNA (cfDNA) has several benefits. Molecular profiling of cfDNA could allow patients to undergo close, longitudinal follow-up that can enable primary diagnosis of extensive disease, optimal choice of treatment, early diagnosis of clinical progression, and identification of secondary resistance mechanisms. In addition, cfDNA analysis offers comprehensive coverage that can help address issues of tumor heterogeneity (Ilie and Hofman, 2016). Collecting and analyzing cfDNA may be particularly advantageous for immunosuppressed patients, as this approach offers a minimally invasive and cost-effective alternative to more traditional molecular profiling methods.

The cfDNA tumor fraction (TF), which is the proportion of tumor molecules that are present in a cfDNA sample, varies greatly across cancer type and between patients, and represents a clinically relevant biomarker (Adalsteinsson et al., 2017; Van Roy et al., 2017; Chen et al., 2019). The detection of tumor-associated single nucleotide variants (SNVs) in the cfDNA can be achieved by using high-depth targeted sequencing methods (Newman et al., 2014; Ishii et al., 2020) or PCR-based methods (Denis et al., 2017). Broader analyses such as whole exome sequencing (WES) at standard depth (∼150 × coverage) are generally informative when the TF is ∼5% to 10% (Cibulskis et al., 2013). Therefore, the estimation of the TF could help guide selection of the appropriate molecular approach for subsequent detection of SNVs in cfDNA.

Recently, a cost-effective approach, ichorCNA11, was established to calculate the TF of cfDNA using low-coverage whole genome sequencing (LC-WGS). This method identifies segmental/arm-level copy number aberrations (CNAs), which enables the TF to be inferred (Adalsteinsson et al., 2017).

The aim of our study was to calculate the TF and determine the CNA profile of cfDNA in immunosuppressed patients or patients with tumors located in immune-privileged sites, such as the central nervous system (CNS) using LC-WGS and ichorCNA. This approach can guide the decision to subsequently perform a targeted PCR-based method, a targeted sequencing, or a WES, based on the circulating tumor content, to provide a more comprehensive molecular analysis of cfDNA in this specific, frail population.

For this purpose, we recruited patients with cancers known to be associated with variable amounts of cfDNA (from low to high) in immunocompetent patients (Mouliere et al., 2018). Specifically, we enrolled untreated immunodeficient patients with non-Hodgkin lymphoma (NHL) (either systemic or localized to the CNS), HIV-positive patients with non-small-cell lung cancer (NSCLC), and immunocompetent patients with glioma.

## Materials and Methods

### Patient Cohort

Patients were enrolled in the IDeATIon project (ClinicalTrials.gov identifier: NCT03706625), a multidisciplinary, multicentric, translational research program that focuses on severe tumors that appear in immunosuppressed patients or in immune-privileged sites such as the CNS. The protocol was approved by the French national IRB (no. 2018-A01099-46) and the “Commission Nationale de l’Informatique et des Libertés” (CNIL no. 918222).

Three types of rare and severe cancers were selected: (1) NHL (either systemic or localized to the CNS) occurring in patients with HIV or in transplant recipients, (2) NSCLC in patients with HIV, and (3) glioma. The inclusion criteria were as follows: (1) male or female adults (age ≥ 18 years old), (2) confirmed histopathological diagnosis of one of the three types of cancer listed above, (3) anti-tumoral treatment-naive (except for some glioma patients), (4) hemoglobin level > 9 g/dl for lung cancer patients and > 7 g/dl for NHL and glioma patients, and (5) written informed consent. From July 2018 to December 2020, 118 patients were enrolled in the IDeATIon project. This study includes 36 of these prospectively enrolled patients (Table 1).

**TABLE 1:** Study cohort description.

	NHL	Lung cancer	Glioma
Number of patients	19	5	12
Mean age of diagnosis (range)	56 (21–76)	61 (58–74)	36 (18–62)
**Sex**			
Male	16 (84%)	5 (100%)	6 (50%)
Female	3 (16%)	0	6 (50%)
**Immune status/localization**			
immunocompromised HIV + /systemic	5 (26.3%)	NA	NA
immunocompromised PTLD/systemic	8 (42.1%)	NA	NA
immunocompromised HIV + /CNS	1 (5.2%)	NA	NA
immunocompromised PTLD/CNS	5 (26.3%)	NA	NA
immunocompromised HIV + /Lung	NA	5 (100%)	NA
Immunocompetent/CNS	0	NA	12 (100%)
**Histology**			
	Monomorphic B-cell PTLD (9)	Non small cell adenocarcinoma (5)	Ganglioglioma (2)
	Polymorphic PTLD (3)		Glioblastoma (4)
	DLBCL (3)		Oligodendroglioma (3)
	Burkitt lymphoma (1)		Astrocytoma(3)
	Marginal zone lymphoma (1)		
	Plasmablastic lymphoma(1)		
	HHV8 + DLBCL,NOS (1)		
**Stage**	**Ann Arbor for systemic NHL**	**TNM classification**	
	Stage I (4)	Stage I	Stage I (2)
	Stage II (0)	Stage II (2)	Stage II (2)
	Stage III (0)	Stage III (1)	Stage III (4)
	Stage IV (9)	Stage IV (2)	Stage IV (4)
**Spreading**	**Extranodal involvement**	**Metastasis**	**Metastasis**
	Yes (10)	Yes (2)	
	No (2)	No (3)	No (12)

*NHL, Non-Hodgkin lymphoma. CNS, central nervous system. DLBCL, diffuse large B cell lymphoma.*

### Spike-In Experiment

Genomic DNA (gDNA) was isolated from two human NSCLC cell lines, H2030 (ATCC^®^ CRL-5914^Tm^) and H1975 (ATCC^®^ CRL-5908^Tm^), and healthy donor blood using a QIAamp DNA Blood Mini Kit (Qiagen). The DNA concentrations were quantified on a Quantus^Tm^ Fluorometer (Promega) using QuantiFluor^®^ ONE dsDNA Dye (Promega). Genomic DNA was sheared to an average size of 150 bp using The Bioruptor^®^ Pico (Diagenode). Fragment size was confirmed on a TapeStation 4200 electrophoresis system (Agilent). To create the spike-in mix, H2030 or H1975 DNA was mixed with healthy donor DNA to a final proportion of 5% or 15%, and the samples were analyzed by LC-WGS.

### Plasma Preparation and cfDNA Extraction

Blood was collected in Cell-Free DNA Collection Tubes (ROCHE), which were then centrifuged for 10 min at 820 *g*. Next, the supernatant was transferred to a fresh tube, centrifuged at 20,000 *g* for a further 10 min, and stored in 1-ml aliquots at −80°C until later extraction. cfDNA was extracted from 5 ml of plasma using the QIAamp Circulating Nucleic Acid Kit (Qiagen) following the manufacturer’s instructions and eluted in a volume of 35 μl. cfDNA was quantified using a Quantus^Tm^ Fluorometer (Promega). The size distribution of the cfDNA was checked on a TapeStation 4200 electrophoresis system (Agilent) using Cell-free DNA ScreenTape.

### LC-WGS and Analysis

Sequencing libraries were prepared from 4 to 10 ng of cfDNA using the SMARTer^®^ ThruPLEX^®^ Tag-seq Kit (Takara Bio). Libraries were quantified using a Qubit^Tm^ dsDNA HS Assay Kit (Thermo Fisher), and the fragment sizes were confirmed using a TapeStation 4200 electrophoresis system (Agilent). Equal amounts of the sequencing libraries were pooled to generate a single sample for sequencing. WGS at an average coverage of 4 × was performed on a Novaseq (Illumina) system using 2 × 150-bp paired-end sequencing. BCL2FASTQ (Illumina) was used to convert bcl files to fastq files. The coverage statistics are summarized in Supplementary Figure 1. Paired-end fastq files were aligned to the hg38 human reference genome using the BWA-MEM algorithm (Li and Durbin, 2009). PCR duplicates were removed, and a Base Quality Score Recalibration (BQSR) was executed with the Genome Analysis Toolkit (GATK) version 4.1 (McKenna et al., 2010) following GATK best practice.

Based on the pre-processed aligned reads, the TF in the cfDNA was calculated using the ichorCNA workflow, as described previously (Adalsteinsson et al., 2017). Briefly, using the readCounter tools from the HMMcopy suite, we calculated the read count coverage of reads with a mapping quality greater than 20 in 1-Mb-window bins across all autosomal chromosomes. The read counts were then normalized to correct for GC content and mappability bias. The copy number analysis and TF prediction were performed using the ichorCNA R software package. LC-WGS sequencing of the healthy donor sample served as the normal WIG. Adjustments were made to the standard ichorCNA settings to account for ploidy (2 or 3) and an initial normal contamination range of 0.1 to 0.99. Genotype count at genomic position on LC-WGS data were performed using bcftools mpileup command (Supplementary Table 1).

### DNA Extraction From Tissue Biopsy

Tumor gDNA was isolated from fresh or formalin-fixed paraffin-embedded (FFPE) diagnostic tissue biopsies using a QIAamp DNA Mini Kit (Qiagen) or a QIAamp DNA FFPE Tissue Kit (Qiagen), respectively, according to the manufacturer’s instructions.

### WES and Tumor Tissue Analysis

Libraries were prepared and hybrid-captured using the SeqCap EZ MedExome Enrichment Kit (Roche) with 200 ng of DNA input. Sequencing was performed on an Illumina Novaseq system with 150-bp paired-end reads. Raw paired-end fastq files were pre-processed using fastp (Chen et al., 2018) for quality control, adapter trimming, and quality filtering. The filtered reads were aligned to the hg38 human reference genome using BWA-MEM (Adalsteinsson et al., 2017). Reads were pre-processed according to GATK best practice. Coverage summary statistics of the consensus coding sequence are shown in Supplementary Figure 2. Somatic SNVs and indels that differed between tumor–normal pairs were called using MuTecT (Cibulskis et al., 2013) and Strelka2 (Kim et al., 2018), respectively. We excluded potential oxidative damage-induced mutations as per Costello et al. (2013) using in-house scripts.

Copy number analysis for tumor biopsy WES was performed using the TitanCNA pipeline (Ha et al., 2014^1^). Briefly, we first identified heterozygous single nucleotide polymorphisms (SNPs) from the matched normal germline blood sample using the Samtools mpileup command and the Exome captured regions bed file and computed read counts for the reference SNPs and alternative genotypes from the tumor biopsy samples. Next, we computed read counts for 50-kb bins using HMMcopy (Ha et al., 2012). Centromeres were filtered based on chromosome gap coordinates obtained from UCSC for the hg38 human reference genome. The read coverage for 50-kb bins across the genome was corrected for GC content and mappability. Finally, TitanCNA was used with default values (except for alphaK = 2500 and minDepth = 20) to generate solutions with one to five clonal clusters, with the initial ploidy set to 2 and 3. The solution with the optimal number of clonal clusters was selected using the minimum S_Dbw validity index (using log ratio and allele ratio) as recommended. Tumor purity estimation was performed using ABSOLUTE (Carter et al., 2012).

### Comparing the LC-WGS Results for the cfDNA and Matched Tumor Biopsies

We performed a log_2_ ratio comparison for 23 patients for whom both LC-WGS cfDNA data and matched tumor biopsy WES data were available (Supplementary Figure 3). We excluded LC-WGS cfDNA segments that did not overlap the tumor biopsy sample. Additionally, we restricted the comparison to segments with cellular prevalence = 1, indicating a higher proportion of tumor cells containing that event. If more than one log_2_ ratio was assigned to the same segments, the median value of the log_2_ ratios in the LC-WGS segments was retained for comparison. Similarity between each cfDNA sample and the matching tumor biopsy was calculated by computing the Spearman correlation coefficient.

### WES and cfDNA Analysis

WES was performed on three cfDNA samples with a detectable TF. The same libraries prepared for LC-WGS were hybrid-captured using a SeqCap EZ MedExome Enrichment Kit (Roche). The sequencing and bioinformatic analysis were performed as described above for tumor tissue, except that variant calling was performed on SNVs only.

### RNA Sequencing and Expression Analysis

RNA was extracted from tumoral tissue using an RNeasy Micro Kit (Qiagen). Libraries were prepared from 500 ng of RNA. After end repair, A-tailing, ligation, and purification, sequencing was performed on an Illumina Novaseq with 150-bp paired-end reads. Reads were aligned to the human hg38 reference genome after an index was generated using STAR v2.7.2 (Dobin et al., 2013) by applying per-sample two-pass mapping. The generated BAM files were pre-processed according to GATK v4.1 RNA-seq best practice (Mark Duplicate, SplitNcigarReads and BQSR), followed by transcript quantification using Kallisto v0.45.0 (Bray et al., 2016). To summarize transcript-level abundance estimates for gene-level analysis, we used *tximport* R packages (Soneson et al., 2015). Gene expression comparison was achieved by simple extraction of the listed genes (ABC and GCB, Supplementary Figure 4C) from total gene expression level, followed by Wilcoxon test to compare the regularized-logarithm transformation (rlog) of the gene count between the two gene sets.

### Statistical Analysis

We used R (version 4.0.0) to perform all statistical analyses and tests. The Wilcoxon test was used to compare ploidy across the exome and LC-WGS data. Copy number variation (CNV) segment tests and *p*-values were computed using Spearman’s test.

## Results

### Cohort Description

The clinical features of the 36 patients included in our cohort are shown in Table 1. Twenty-four patients were immunodeficient, namely, 19 who had NHL and 5 who had NSCLC. Among the patients with NHL, 6 were HIV-positive [1 with primary central nervous system lymphoma (PCNSL) and 5 with systemic NHL] and 13 were organ transplant recipients (5 with PCNSL and 8 with systemic NHL). All five patients with NSCLC were HIV-positive. The other 12 patients in the cohort were immunocompetent and had glioma. Biological material, including blood and tumor tissue samples, was collected at the time of diagnosis and prior to any anti-tumoral treatment (except for some glioma patients).

### Estimation of the TF in the cfDNA

To establish the LC-WGS protocol and ensure reproducibility, we first performed a spike-in experiment by mixing gDNA from two lung cancer cell lines (H2030 and H1975) with known genomic profiles with DNA from a healthy donor at different ratios. We found that a detectable TF using our method was a TF > 5% (Supplementary Figure 1). Following LC-WGS and bioinformatics analysis, the TF in cfDNA from 24 immunodeficient patients with cancer (19 NHL and 5 NSCLC-HIV) and 12 immunocompetent patients with glioma was determined. We prioritized ichorCNA solutions with a ploidy of 2, as our cohort did not exhibit high-copy genome amplification, and subjected the CNV profiles to manual review to account for bias introduced to bin log_2_ ratio values after GC content correction.

**FIGURE 1:**
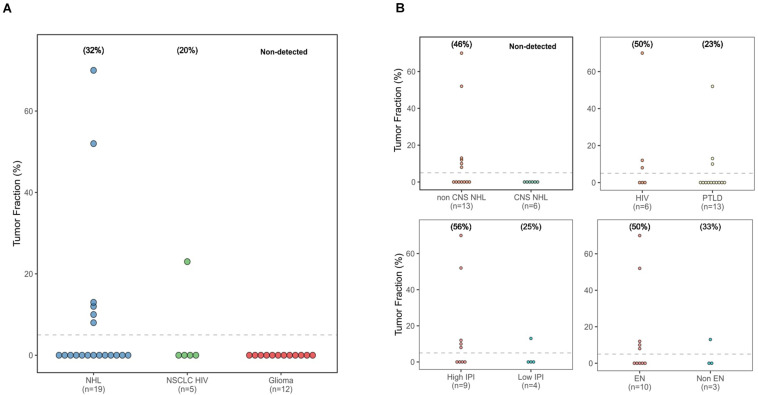
Tumor fraction dot plots. **(A)** All three cancer types studied. **(B)** Based on non-Hodgkin lymphoma location, immunodeficiency type, nodal location, and International Prognostic Index group.

The calculated TF values ranged from 8 to 70% (Figure 1A). Thirty-two percent of immunodeficient NHL patients (6/19) and 20% of HIV-positive NSCLC patients (1/5) had a TF greater than 5%. However, no tumor-derived cfDNA was detected in any of the glioma or PCNSL patients. The estimated TF for the HIV-positive patient with NSCLC was 23%; this patient had metastatic cancer (T4 N3 M1c, stage 4B disease).

Next, we compared TF values across different groups of patients with NHL, based on localization, International Prognostic Index (IPI) score, and type of immunodeficiency (Figure 1B). Six of the NHL patients with detectable TF (TF = 8%, 10%, 12%, 13%, 55%, and 70%) had exclusively systemic localization. In contrast, the TF was below the limit of detection of all the patients with PCNSL. Five out of the six patients with a TF value > 5% had extranodal manifestations of disease. No difference was observed in terms of type of immunodeficiency. Finally, five out of nine patients with a high IPI score (2 or 3) exhibited detectable levels of tumor cfDNA, whereas only one in four with a low IPI score (0 or 1) had a detectable TF.

### Copy Number Alteration Profile Concordance Between Tumor Biopsies and cfDNA

**FIGURE 2:**
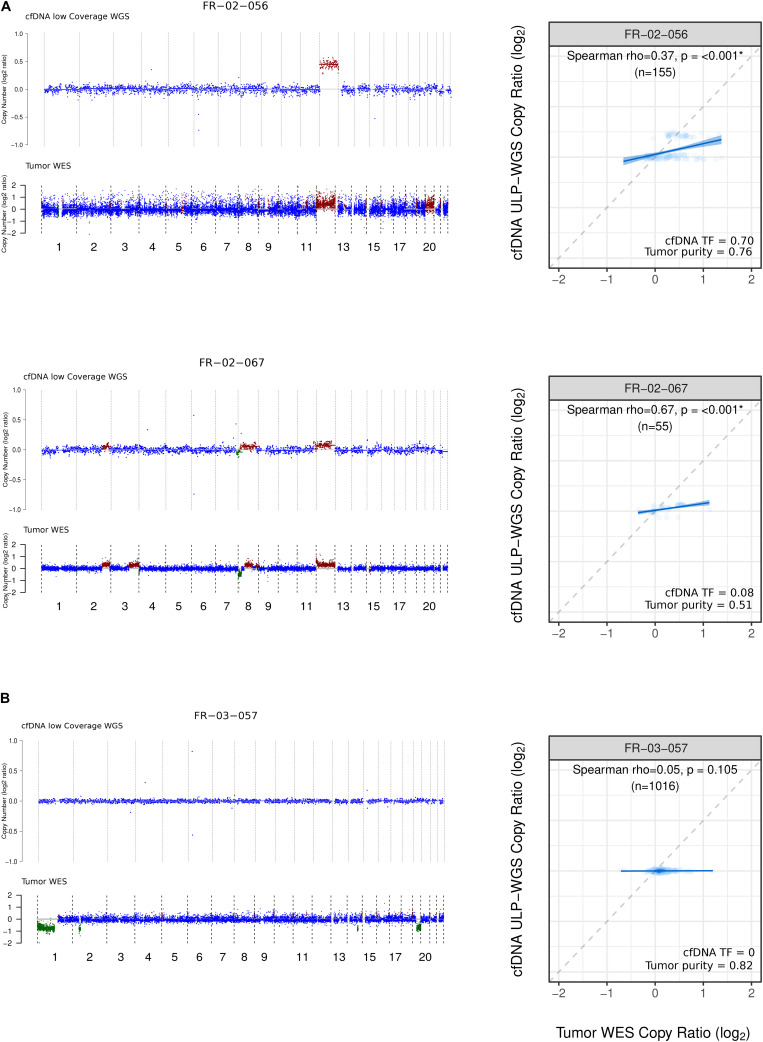
Copy number profile and comparison from LC-WGS cfDNA and WES tumor biopsy. **(A)** Left track shows genome-wide copy number from two ID-NHL patients (FR-02-056 and FR-02-067 with the following median coverage for cfDNA 4.24X, 3.49X and for WES tumor biopsy 216X, 211X, respectively). Right track shows cfDNA tumor fraction (TF) computed using ichorCNA and Tumor purity calculated using ABSOLUTE. **(B)** Left track shows genome-wide copy number from a 1p19q codeleted glioma patient FR-03-057 with the following median coverage for 4.30X for cfDNA and 166 X for WES tumor biopsy. Right track shows Spearman rank correlation between LC-WGS cfDNA and WES tumor biopsy (see *Materials and Methods*).

Next, we compared the CNA profiles for the cfDNA samples as established by LC-WGS and ichorCNA with the CNA profiles deduced from WES of matched tumor biopsies (100× to 200×, *n* = 23). These analyses involve comparison of the normalized log_2_ copy ratios between the two sample types from same patients. We found that the Mb-scale copy numbers correlated well in samples with a TF > 5% (ranging from ρ = 0.34 to ρ = 0.81; *p* < 0.001, Figure 2A), while there was no significant correlation for patients with undetectable TFs (ρ = −0.31 to ρ = 0.08; Figure 2B, Supplementary Figure 3).

### Copy Number Alteration Profile Analysis of cfDNA

To evaluate whether cfDNA can serve as a proxy for tumor biopsies in cancer analyses, we analyzed the CNA profiles of cfDNA from patients with a TF > 5%.

**FIGURE 3:**
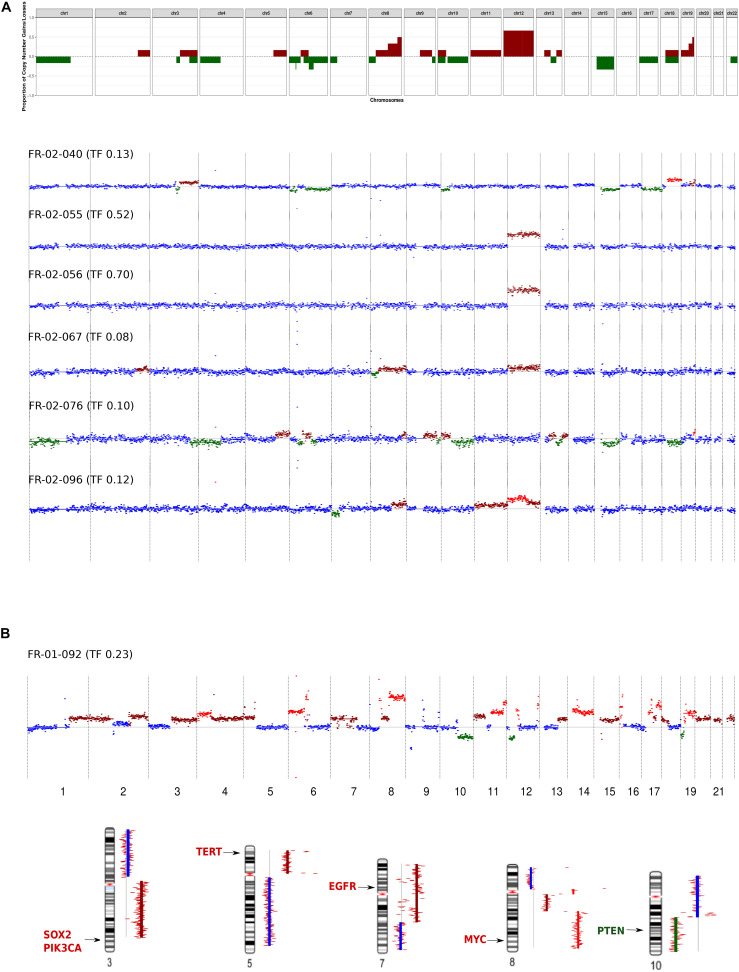
Genome-wide copy number alteration (CNA) landscape in patients with a tumor fraction >5%. **(A)** Cell-free DNA (cfDNA) results from six immunodeficient non-Hodgkin lymphoma patients. Top, frequency of copy number variation (CNV) regions detected by low-coverage whole-genome sequencing of cfDNA. Bottom, CNV pattern for each patient. The plots show the CNV log_2_ ratios between the cfDNA sample and the healthy donor (HD). Neutral copy numbers are shown in blue, copy number gains are shown in dark red, copy number amplifications are shown in red, and copy number losses are shown in green. **(B)** cfDNA results from the non-small-cell lung cancer (NSCLC) patients infected with human immunodeficiency virus. Top, CNV pattern. Bottom, genes affected by CNA known to be associated with NSCLC, and their chromosomal locations.

In the HIV-positive patient with NSCLC, chromosomal gains were identified for several genes known to be associated with NSCLC, such as *SOX2* (3q), *TERT* (5p), and *EGFR* (7q). Furthermore, we found amplification of *MYC* and heterozygous deletion of the *PTEN* tumor suppressor, located at 8q and 10q, respectively (Figure 3B).

In the NHL group, at the chromosomal level, chromosome 12 gain was the most frequent event (encountered in 4/6 patients), followed by chromosome 15 loss (detected in 2/6 patients). These CNAs were mutually exclusive in our cohort (Figure 3A). Focal chromosomal alterations were also detected, including 8q24 gain and 6p22, 6q21 loss, which were identified in 3/6 and 2/6 patients, respectively. These findings were compared to known cancer-associated somatic CNAs seen in patients with NHL (Lopez et al., 2019; Cascione et al., 2020) or NSCLC (Spoerke et al., 2012; Qiu et al., 2017).

The NHL patients in our cohort exhibited two distinct CNA profiles. The first group included patients [three patients with diffuse large B-cell lymphoma (DLBCL) and one patient with Burkitt lymphoma] who exhibited gain of chromosome 12, which is where *STAT6*, *MDM2*, and *BCL7A* are located. The second group (two patients with DLBCL) exhibited neutral chromosome 12 copy numbers but had gains in a region harboring the HLA locus, *PRDM1*, and *POLG1* (Supplementary Figure 4A). Interestingly, patient FR-02-40 harbored alterations in genes associated with the activated B-cell (ABC) DLBCL subgroup (Testoni et al., 2015; Schmitz et al., 2018), such as *PIK3CA*, *NFKBIZ*, *BCL2*, and *SPIB*.

To confirm whether this patient belonged to the ABC molecular subgroup, we analyzed somatic mutations from WES analysis of a biopsy of the patient’s tumor. We identified mutations in the tumor suppressor gene *TBL1XR1* and the proto-oncogene *PIM1* (Supplementary Figure 4B). In addition, RNA-seq expression analysis of the matched tumor tissue revealed overexpression of ABC-associated genes (Alizadeh et al., 2000; Rosenwald et al., 2002) (Supplementary Figure 4C). Finally, immunohistochemistry analysis using the Hans algorithm classified patient FR-02-40 as non-GCB.

### WES of cfDNA

The availability of sufficient tumor-derived cfDNA material allowed us to generate WES data (median coverage of 200×) on three of the cases with LC-WGS and tumor biopsy exome sequenced. We detected shared coding somatic SNV in cfDNA and tumor biopsy in FR-02-040-BP (16%), FR-02-056-LTT (28%), and FR-01-092-PA (83%). Across the common mutations, we identified mutations of known-driver genes in NSCLC as *TP53* and *MAP2K1* (Wislez et al., 2021) and recurrent mutated genes in NHL as *MYC, TBL1XR1, IGLL5, GNA13*, and *PIM1.* We found a strong correlation between the variant allele frequency (VAF) on cfDNA and the somatic VAF in tumor tissue biopsy (Pearson test, ρ = 0.7–0.78, all *p*-value < 0.001, Supplementary Figure 4). Notably, the median VAFs of shared SNV were larger as the estimated TFs were higher (Supplementary Table 2). In addition to somatic variant analysis, we estimated ploidy and purity in cfDNA WES data. Computed tumor purity on cfDNA was comparable to TFs calculated using LC-WGS (Supplementary Table 2).

## Discussion

Studies have shown that LC-WGS and ichorCNA analysis can accurately detect low fractions of circulating tumor DNA in blood plasma (Adalsteinsson et al., 2017; Chen et al., 2019). Calculating the TF in the cfDNA can help select a method with appropriate sensitivity for performing a comprehensive molecular analysis. Using CNAs to calculate the TF is particularly relevant, as the vast majority of cancers harbor arm-level somatic CNAs (Beroukhim et al., 2010).

Our pilot study is the first to calculate TFs and characterize somatic CNA profiles for cfDNA using LC-WGS and ichorCNA in immunosuppressed patients (HIV-positive or transplant recipients) with NHL (systemic or CNS-localized) or lung cancer. Immunocompetent patients with glioma were also included, given the location of the tumor in an immunosuppressive environment, and, for the purposes of this pilot study, were expected to exhibit a low TF in the blood (Bettegowda et al., 2014).

A TF was detectable in 29% (7/24) of the immunodeficient patients in our cohort, with a TF range of 8% to 70%.

No TFs or CNAs were detected in PCNSL and glioma patients. The failure to detect a TF in these patients was presumably caused by insufficient ctDNA levels in the blood that were below the limit of detection for ichorCNA. Indeed, the lower limit of ichorCNA sensitivity for detecting the presence of tumor is 3% (Adalsteinsson et al., 2017). Glioma-associated mutations have been detected in liquid biopsies using highly sensitive droplet digital PCR (Muralidharan et al., 2021) or by analyzing cerebrospinal fluid (CSF) instead of plasma (Mouliere et al., 2018), suggesting that it could be valuable to test LC-WGS on CSF cfDNA in PCNSL patients. Our results support the use of targeted molecular analyses with high sensitivity when the TF is not detected or is very low.

In contrast, patients with a detectable TF should be selected for further larger-scale sequencing methods, such as WES, for which the calculated TF could help guide calibration of the sequencing depth.

One immunosuppressed lung cancer patient included in our study had a TF of 23%. This patient had non-small-cell adenocarcinoma, was HIV-positive, and had advanced metastatic disease (T4 N3 M1c, stage 4B disease). The CNA profile derived from the tumor tissue biopsy taken from this patient correlated well with the findings from the corresponding liquid biopsy (ρ = 0.81). Moreover, the CNA profile was consistent with patterns that are typically observed in patients with NSCLC (Chen et al., 2019; Raman et al., 2020). Determining the CNA profile from cfDNA could constitute an attractive method to support histological subtyping of lung cancer patients, as suggested by Raman et al.

In our study population, the immunodeficient NHL patient group had the highest proportion of patients with detectable aberrations (6/19, 32%). The proportion of patients with detectable cancer-derived cfDNA in this group was lower than that described in the literature for NHL patients. Bohers et al. identified tumor-associated mutations in the cfDNA in 19/30 patients (63%), and Rossi et al., in 20/30 patients (66.6%) (Rossi et al., 2017; Bohers et al., 2018). However, these two studies focused on targeted detection of somatic SNVs in recurrently mutated cancer genes using high-throughput targeted sequencing. It has been shown that a CNA is > 20 times more likely to be detected by LC-WGS than by arrays (Zhou et al., 2018).

Interestingly, 5/6 NHL patients with a TF > 5% had extranodal manifestations and a high IPI score. Given that our patients were untreated at the time of liquid biopsy, our results agree with a study concluding that ctDNA is a promising biomarker for identifying NHL patients at high risk of treatment failure and at risk of recurrence before clinical evidence of disease relapse (Roschewski et al., 2015).

The potential utility of cfDNA as a biomarker and as a way to molecularly characterize tumors is supported by the concordance between the cfDNA and tumor tissue CNA profiles. Despite the low number of patients with cfDNA WES data, VAF comparison and purity estimation collectively suggests an accurate sensitivity of TF estimation using LC-WGS/IchorCNA. Surprisingly, the strongest correlation was found in the patient with a TF of 23% and not in the patient with a TF of 70%. This may be attributable to the fact that several CNA events were detected in the patient with the lower TF, while only chromosome 12 gain was found in the patient with the higher TF. However, this patient harbors coding variants with high VAFs in cfDNA (median VAF = 0.28, Supplementary Table 2).

The CNA profiles derived from the cfDNA from the DLBCL subgroup are consistent with patterns that are usually observed in DLBCL and have been previously detected by analyzing tumor tissue (Testoni et al., 2015). However, based on events observed in the cfDNA CNA profiles, we were further able to distinguish two distinct groups of patients. These two groups (chromosome 12 gain or neutral copy number) correlate with the ABC and GCB gene expression subgroups (Schmitz et al., 2018). Furthermore, mutations in genes corresponding to one subgroup or the other were found in the matched tumor tissue. These results should be confirmed in a larger cohort.

## Conclusion

Our pilot study showed, for the first time, that ctDNA can be detected by LC-WGS/ichorCNA in the plasma of immunosuppressed patients with advanced NHL or lung cancer. This cost-effective method accurately calculated the TF and determined the CNA profile from the cfDNA and can be used to guide the choice of molecular method for further larger-scale investigations of cfDNA. Applying this method to a larger cohort of immunodeficient patients, as well as an immunocompetent cohort, may help shed light on cancer pathogenesis, the genomic mechanisms by which tumor cells acquire treatment resistance, and the response to treatment in this specific population. Liquid biopsy can be particularly useful in immunosuppressed patients, who are at higher risk for complications during tissue biopsy. Using this approach, molecular information from ctDNA can be collected before and during treatment to help improve patient care. Future analyses could help elucidate the direct impact that immune system impairment has on the circulating TF in these patients.

## Members of the IDEATION Study Group

Ahmed Idbaih, Noureddine Balegroune, Amélie Guihot, Ioannis Theodorou, Agusti Alentorn, Isabelle Brocheriou, Anne-Geneviève, Damien Roos Weil, and Alberto Picca.

## Data Availability Statement

The data presented in the study are deposited in the European Nucleotide Archive repository, accession number PRJEB43455, link: https://www.ebi.ac.uk/ena/browser/view/PRJEB43455.

## Ethics Statement

The studies involving human participants were reviewed and approved by the IRB (no 2018-A01099-46) and “Commission Nationale de l’Informatique et des Libertés” (CNIL no 918222). The patients/participants provided their written informed consent to participate in this study.

## Author Contributions

AB and KL processed the experimental data, performed the analysis, drafted the manuscript, and designed the tures. AB and SJ carried out the experiments. KL and P-YB performed the bioinformatic analyses. FCo, JD, and J-ML were involved in planning and supervised the work and the manuscript draft. J-PS and BA conceived of the project IDEATION study. MV is the project manager of IDEATION study. EG helped for experimental process. FCh and FB are anatomopathologists involved for selection of tumor tissue. MB, MT, FD, SC, VL, and JC aided in interpreting the results and worked on the manuscript. All authors discussed the results and commented on the manuscript.

## Conflict of Interest

The authors declare that the research was conducted in the absence of any commercial or financial relationships that could be construed as a potential conflict of interest.

## Publisher’s Note

All claims expressed in this article are solely those of the authors and do not necessarily represent those of their affiliated organizations, or those of the publisher, the editors and the reviewers. Any product that may be evaluated in this article, or claim that may be made by its manufacturer, is not guaranteed or endorsed by the publisher.

## Footnotes

1
https://github.com/gavinha/TitanCNA
