# Supplementary material for: Low-Coverage Whole Genome Sequencing of Cell-Free DNA From Immunosuppressed Cancer Patients Enables Tumor Fraction Determination and Reveals Relevant Copy Number Alterations
Source: Front Cell Dev Biol. 2021 Aug 3;9:661272. doi: 10.3389/fcell.2021.661272 (PMC8369887; doi:10.3389/fcell.2021.661272)
Supplement: Supplementary file 1 [file Data_Sheet_1.docx]

**Supplementary Data**


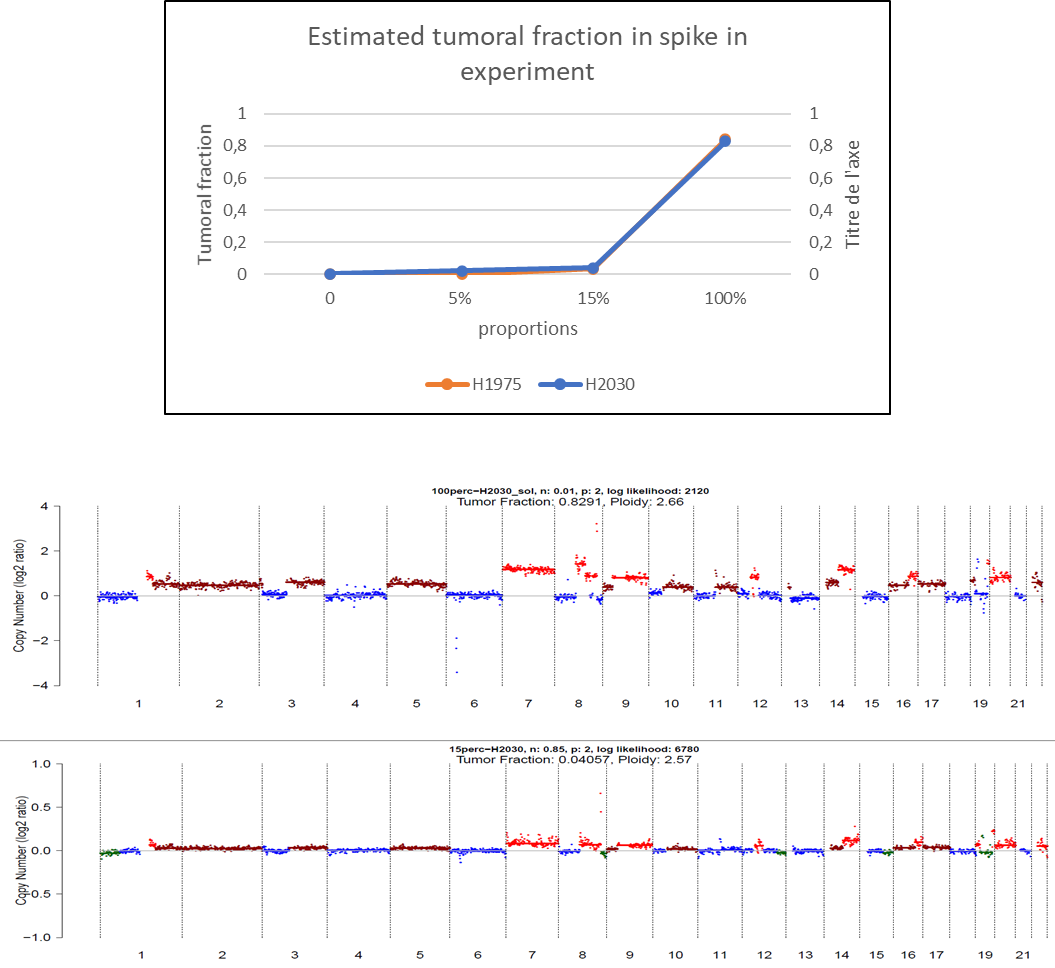


**Figure 1** : Spike in experiment. Spike in experiment

The estimated tumor fraction is proportional to the tumor DNA spiked. CNAs profile were similar at all proportions. However, the estimated tumor fraction was somewhat lower than the actual spike-in levels. We hypothesize that there were loses in tumor DNA during the wet lab manipulations. Also, the tumor fraction estimation may be more accurate for cell lines with a higher proportion of copy number gains, as gains are easier to detect with LC-WGS.


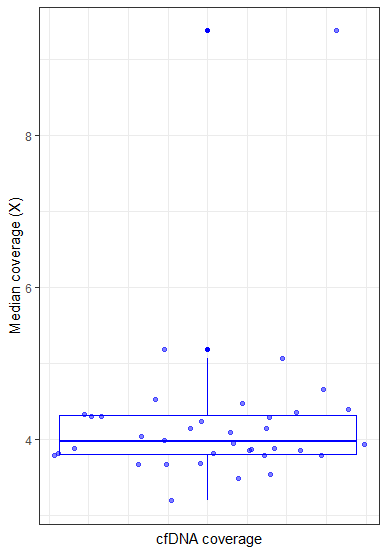


**Figure 2** LC-WGS coverage summary boxplot (n=36)


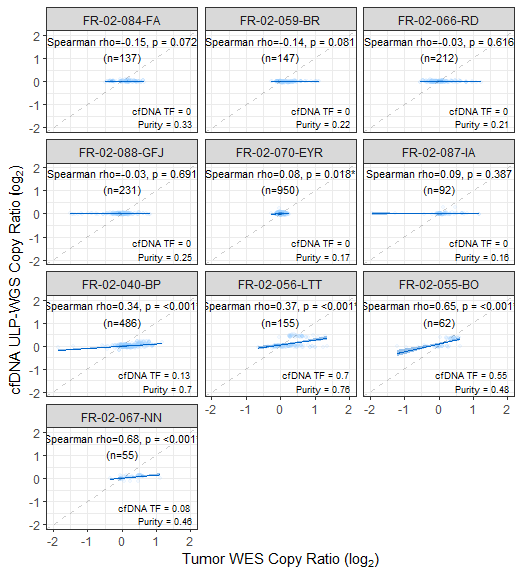


**A**


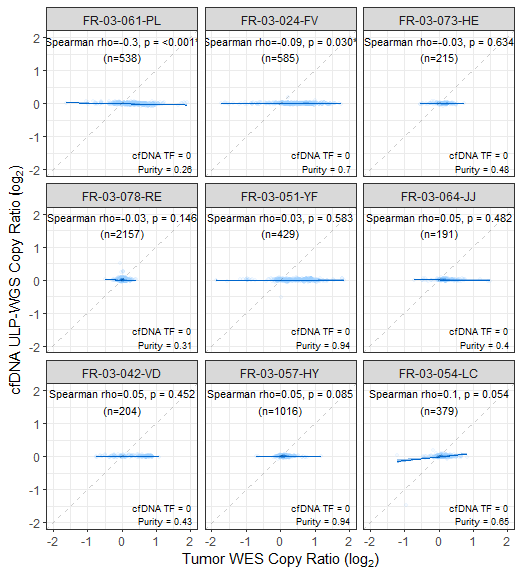
**B**


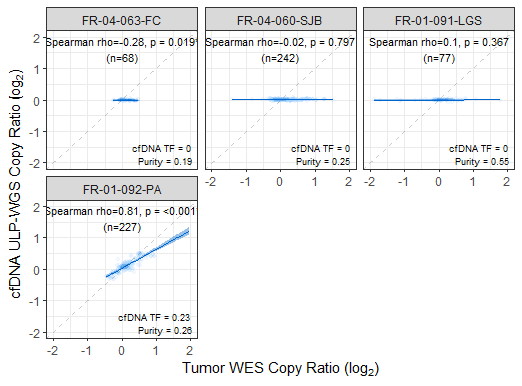


**D**

**C**


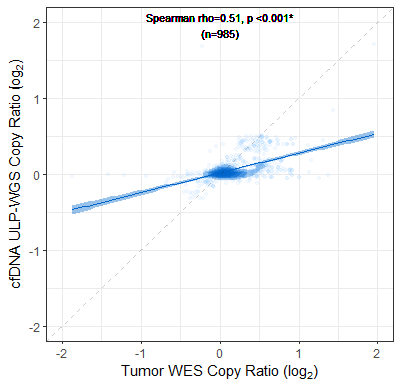


**D**

**Figure 3.** Assessing the similarity between each LC-WGS cfDNA sample and the WES matching tumor biopsy. **A.** for Glioma patients (n=9), **B.** for NHL (n=10), **C.** NSCLC (n=4). **D.** for patients with TF >=5 % (n=5). See method for spearman comparison.


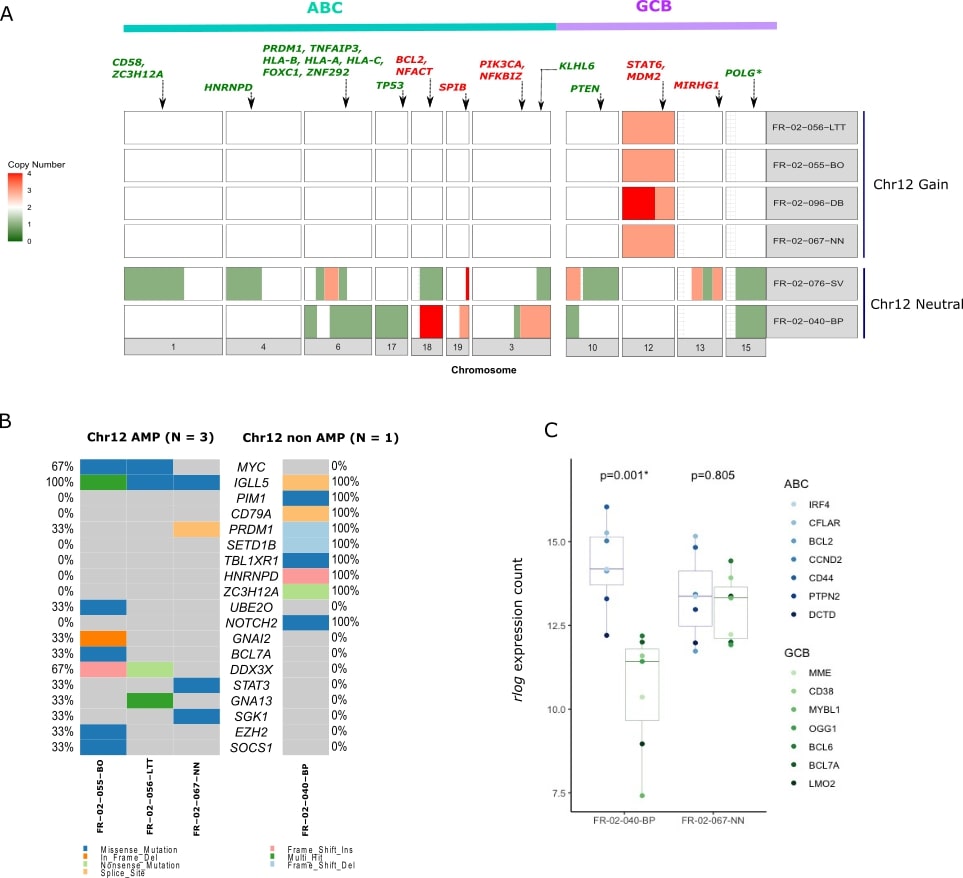


**Figure 4.** ABC/GCB genetic alterations enriched in ID-NHL studied patient. A CNV profile in ID-NHL ordered by known ABC/GBC gene CNA and chromosome 12 statute based on LC-WGS cfDNA. B Mutation found in known most frequent mutated genes in DLBCL and BL from four ID-NHL studied patient with WES tumor biopsy. C Gene expression comparison of ABC and GCB associated gene in two ID-NHL (DLBCL) studied patients.


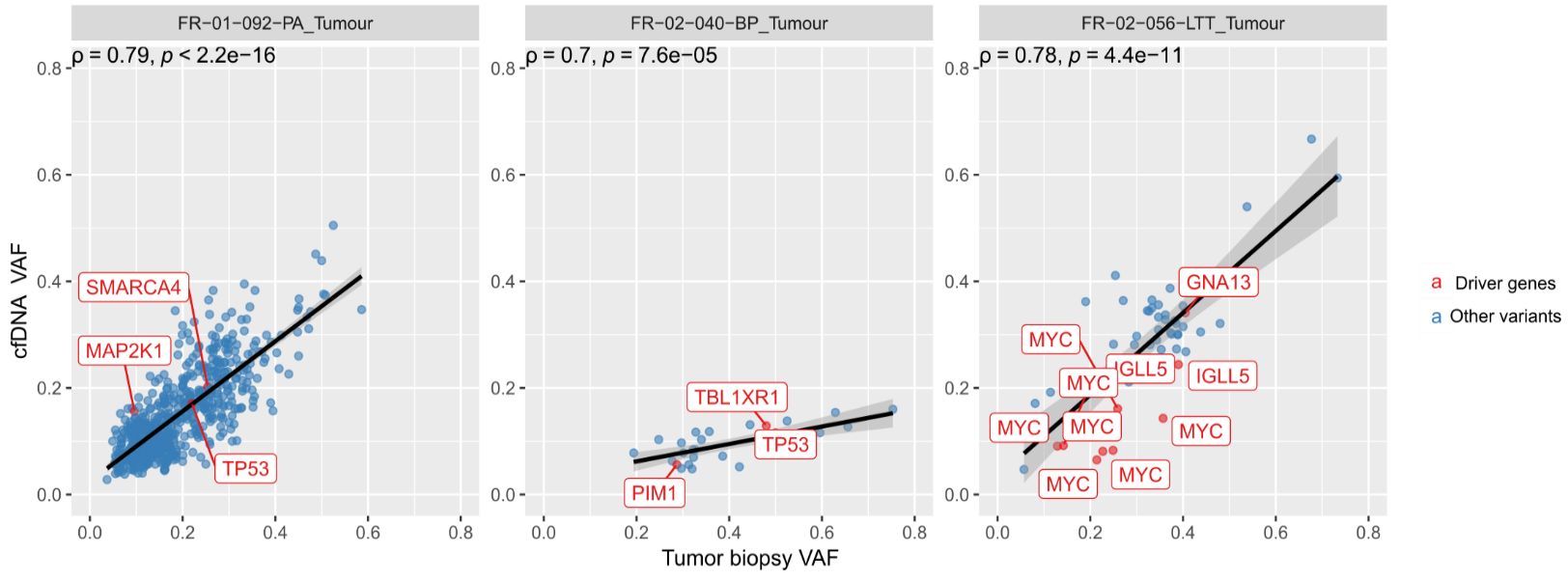


**Figure 5.** Variant allele frequency correlation of cfDNA and Tumor biopsy using WES data.

| patients | LC-WGS estimated  Tumor Fraction | nb coding shared  variant (gene) | LC-WGS_alt_count | LC-WGS_Depth | Tumor biopsy  VAF |
| --- | --- | --- | --- | --- | --- |
| FR-02-056-LTT | 70% | 1 (KNDC1) | 3 | 4 | 0.372 |
| FR-01-092-PA | 23% | 1 (TEAD2) | 8 | 3 | 0.443 |
| FR-02-055-BO | 52% | 0 |  |  |  |
| FR-02-040-BP | 13% | 0 |  |  |  |
| FR-02-067-NN | 8% | 0 |  |  |  |
| FR-01-091-LGS | 0% | 0 |  |  |  |
| FR-02-059-BR | 0% | 0 |  |  |  |
| FR-02-066-RD | 0% | 0 |  |  |  |
| FR-02-070-EYR | 0% | 0 |  |  |  |
| FR-02-084-FA | 0% | 0 |  |  |  |
| FR-02-087-IA | 0% | 0 |  |  |  |
| FR-02-088-GFJ | 0% | 0 |  |  |  |
| FR-02-089-DT | 0% | 0 |  |  |  |
| FR-03-024-FV | 0% | 0 |  |  |  |
| FR-03-042-VD | 0% | 0 |  |  |  |
| FR-03-051-YF | 0% | 0 |  |  |  |
| FR-03-054-LC | 0% | 0 |  |  |  |
| FR-03-057-HY | 0% | 0 |  |  |  |
| FR-03-061-PL | 0% | 0 |  |  |  |
| FR-03-064-JJ | 0% | 0 |  |  |  |
| FR-03-073-HE | 0% | 0 |  |  |  |
| FR-03-078-RE | 0% | 0 |  |  |  |
| FR-03-093-CS | 0% | 0 |  |  |  |
| FR-04-060-SJB | 0% | 0 |  |  |  |
| FR-04-063-FC | 0% | 0 |  |  |  |

**Table 1.** LC-WGS genotype count summary

**Table 2.** cfDNA WES somatic variant calling and purity analysis summary results.

| **Data type** |  | **FR-02-040-BP** | **FR−01−092−PA** | **FR-02-056-LTT** |
| --- | --- | --- | --- | --- |
| **LC-WGS** | TF LC-WGS | 0,13 | 0,23 | 0,7 |
|  |  |  |  |  |
| **cfDNA WES** | Purity WES cfDNA | 0,21 | 0,21 | 0,66 |
|  | VAF median | 0,08 | 0,12 | 0,28 |
|  | Coding SNV number | 37 | 660 | 67 |
|  | Tumor shared SNV (%) | 26 (16%) | 633 (83%) | 42 (28%) |
|  |  |  |  |  |
| **Tumor biopsy WES** | Purity WES Tumor | 0,7 | 0,26 | 0,76 |
|  | Coding SNV number | 160 | 763 | 150 |
